# The Ready Method in Cases of Choking

**Published:** 1857-05

**Authors:** 


### *The Ready Method in Cases of Choking.*

By Marshall Hall, M. D.

Death in choking is the result of a diastaltic spasmodic closure of the glottis.

Nothing can be done in this stage of the accident, except, 1, to endeavor, by introducing the finger into the fauces, to induce vomiting; 2, to introduce something *like a bougie* into the œsophagus (a firm scroll of linen being the readiest); or, 3, to adopt a measure, which I adopted on an emergency, with immediate success, some years ago.

A little boy, eating some fowl in haste, attempted to swallow too large a morsel, and was choked; I ran to him, placed him between my knees, one knee (the right) pressing firmly on the stomach, the other on the back; I then placed one hand (the left) on the back part of the thorax, whilst I gave a firm blow with the other on the sternum. In an instant, I had the joy of seeing the morsel of chicken expelled with force to a considerable distance; and all was safe!

But supposing all these efforts to fail. What then is to be done?

In the midst of the asphyxia induced by the closure of the glottis, the excito-motor power fails, and the larynx is no longer spasmodically closed; and now the 'Ready Method' may be adopted, with the effect of sustaining life, until such a bougie is made as shall be effectual in pushing down the morsel of food or other object in the pharynx or œsophagus.

A *firm* scroll of cotton or linen, when imbued with grease, made from a sheet, a window blind, or curtain, may then be made, not in too great haste, and be boldly passed into the œsophagus.

The morsel of food is generally lodged in the pharynx or *upper* part of the œsophagus, and, when found lower down, ceases to excite reflex action of the larynx; and breathing is, therefore, possible.

A thin bent tallow-candle, or a piece of firmish *cord* (taken from the window frame), might answer the purpose of the bougie.

The 'Ready Method' procures us the *time* necessary for obtaining or preparing *any* of these means, and for giving full directions to the assistants. In performing it, a little brisk movement may be adopted in pronation, and in making dorsal pressure, which may, if not at first, eventually dislodge the foreign body.

I need scarcely suggest that this last measure should also be enforced in cases of a foreign body inhaled into the larynx both *before* and *after*

tracheotomy, with the addition of a firm blow, with the open hand, on the back.

*January*, 1857.

P. S.—I have been informed of the case of a patient, dying from the influence of *chloroform*, in one of the London hospitals, having been rescued by the 'Ready Method.' Surely it should be published in all details. —*Ibid.*

